# ENACT study: What has helped health and social care workers maintain their mental well‐being during the COVID‐19 pandemic?

**DOI:** 10.1111/hsc.13992

**Published:** 2022-09-06

**Authors:** Nicola Cogan, Chloe Kennedy, Zoe Beck, Lisa McInnes, Gillian MacIntyre, Liza Morton, Gary Tanner, Jacek Kolacz

**Affiliations:** ^1^ School of Psychological Sciences & Health University of Strathclyde Glasgow UK; ^2^ School of Social Work & Social Policy University of Strathclyde Glasgow UK; ^3^ Traumatic Stress Research Consortium (TSRC) Kinsey Institute, Indiana University Bloomington Indiana USA

**Keywords:** adaptation, COVID‐19, health and social care workers, help‐seeking, mental health, peer support, well‐being

## Abstract

A growing body of research has highlighted the adverse impact of COVID‐19 stressors on health and social care workers' (HSCWs) mental health. Complementing this work, we report on the psychosocial factors that have had both a positive and negative impact on the mental well‐being of HSCWs during the third lockdown period in Scotland. Using a cross‐sectional design, participants (*n* = 1364) completed an online survey providing quantitative data and free open‐text responses. A multi‐method approach to analysis was used. The majority of HSCWs were found to have low well‐being scores, high levels of COVID‐19 stress, worry, burnout and risk perception scores and almost half of HSCWs met the clinical cut‐off for acute stress (indicative of PTSD). HSCWs with higher scores on adaptive coping strategies and team resilience reported higher scores on mental well‐being. HSCWs were significantly more likely to seek informal support for dealing with personal or emotional problems compared to formal supports. Barriers to formal help‐seeking were identified including stigma and fear of the consequences of disclosure. HSCWs mostly valued peer support, workplace supports, visible leadership and teamwork in maintaining their mental well‐being. Our findings illuminate the complexity of the effects of the COVID‐19 pandemic on HSCWs' well‐being and will inform future intervention development seeking to increase positive adaptation and improve staff well‐being. Addressing barriers to mental health help‐seeking among HSCWs is essential. The implications emphasise the importance of lessons learned across health and social care contexts, planning and preparedness for future pandemics.

**What is known about the topic?**

- Health and social care staff have experienced substantial deterioration in their mental health since the onset of the pandemic.
- A number of COVID‐19‐related challenges have been reported including increased worry and risk of infection and a need to rapidly transform how services have been delivered.
- The importance of adaptive coping and team resilience have been well documented, although the relationship between these and formal and informal help‐seeking is less well understood.

**What does this paper add?**

- Both health and social care workers are at risk of poor mental well‐being, burnout and post‐traumatic stress as a result of the COVID‐19 pandemic.
- A combination of peer support, adaptive coping and team resilience may offer enhanced protection against COVID‐19 stressors moving forward.
- Staff highlighted numerous barriers to formal help‐seeking, including stigma and fear of disclosure, and expressed a preference for informal help‐seeking.

## 1 INTRODUCTION

Rapid research world‐wide has reported on the unprecedented COVID‐19‐related challenges facing health and social care workers (HSCWs) and how these prompted substantial rates of mental health deterioration (Badahdah et al., 2020; Barzilay et al., 2020; Cag et al., 2021; Cogan et al., 2022; De Kock et al., 2021; Fang et al., 2021; Feinstein et al., 2020; Mascayano, et al., 2022; Mehta et al., 2021; Moitra et al., 2021; Odani et al., 2022; Rana et al., 2020; Vanhaecht et al., 2021; Young et al., 2021; Ziarko et al., 2022). Work published during the first wave of COVID‐19 largely focused on healthcare workers, reporting clinically significant symptoms of stress, depression, anxiety, insomnia, burnout and psychological distress (García‐Fernández et al., 2020; Inchausti et al., 2020; Nyashanu et al., 2022) and emphasised the importance of safeguarding staff mental health (De Brier et al., 2020; Jordan et al., 2021; Lake et al., 2022; Nyashanu et al., 2022; Spoorthy et al., 2020). Research within the UK has reported that PTSD rates, for healthcare workers, in NHS intensive care units were found to be double those in combat veterans (Greenberg et al., 2021) and 58% of HSCWs in a recent survey met the threshold for a clinically significant disorder across occupational groups and settings (Greene et al., 2021). Findings from a further UK based study reported that applied health professionals and social workers were significantly at risk of well‐being decline, stress and burnout prior to the pandemic due to intense workload pressures and demands (McFadden et al., 2021).

A range of personal and professional COVID‐19‐related challenges which pose risks to HSCWs' mental health have been reported including prolonged waiting lists, adapting to new technologies, worry over staff infection rates, exposing family members to risks of infection and concerns for their own mental and physical well‐being (Cogan et al., 2022; Currie et al., 2020; Greenberg, 2020; Shanafelt et al., 2020; Zaka et al., 2020). Such concerns may negatively affect HSCWs' mental well‐being by igniting fear of COVID‐19 and hesitancy to engage in direct patient contact (Sperling, 2021; Yıldırım et al., 2020).

HSCWs have reportedly been required to develop and navigate new ways of working, support colleagues, and manage a more demanding workload at a reduced organisational capacity due to staff absence and/or redeployment (Billings et al., 2021; Pereira‐Sanchez et al., 2020). The British Medical Association (2020) outlined that these workforce shortages have had a corrosive impact on morale, due to unmanageable workloads and inability to provide their preferred quality of care. In addition, rapid adaption of service models from face‐to‐face to tele‐health (Whaibeh et al., 2020), may also contribute to the perceived inability to provide the usual quality of care resulting in moral injury (Williamson et al., 2020; Zerach & Levi‐Belz, 2022).

Social workers and social care staff reported similar pressures including dealing with service users with complex needs and the rapid transition to virtual support (Ashcroft et al., 2021). Social workers, in 607 responses to an online survey, also highlighted some of the challenges of practicing ethically during the pandemic including maintaining trust, dignity, privacy and autonomy in remote relationships as well as deciding how to allocate scarce resources (Amadasun, 2020; Banks et al., 2020). Mental health workers, who have also provided well‐being support to HSCWs during this pandemic have also reportedly experienced the adverse impact of COVID‐19‐related stressors on their own mental well‐being, including vicarious trauma (Billings et al., 2021; Cogan et al., 2022), as well as concerns about service users thought to be disproportionately affected by the pandemic such as those experiencing loneliness, abuse and/or family conflict (Johnson et al., 2021). Together, growing research highlights the adverse impact of COVID‐19 stressors across occupational groups within the health and social care sector (Riedel et al., 2022).

### 1.1 Adapting in the face of adversity

There has been an increasing focus on understanding what may help HSCWs adapt to the adversities associated with COVID‐19 and help to protect their mental well‐being moving forward (Benham et al., 2022; Jordan et al., 2021; McFadden et al., 2021; Ortiz‐Calvo et al., 2022; Portugal et al., 2022). Given the ongoing nature of the pandemic a shift from reactive crisis coping toward proactively embedding protective measures is essential. The protective concept of resilience, defined as the ability to cope and/or positively adapt to adversity (Connor & Davidson, 2003; Masten, 2018), has been found to mitigate or prevent severe stress and maintain mental health via a resilient mindset and coping behaviours, such as support seeking and meaning making (Barzilay et al., 2020; Bonanno, 2004; Duan et al., 2015; Hao et al., 2015; Hu et al., 2015; Labrague, 2021; Lissoni et al., 2020; Tahara et al., 2021; Willis & Burnett Jr., 2016; Yildirim, 2019). Studies across different countries have shown that resilience reduces the adverse impact on mental health associated with COVID‐19 stressors, specifically PTSD, anxiety, burnout and depression symptoms (Li et al., 2022; Luceño‐Moreno et al., 2020; Riehm et al., 2021; Soares et al., 2022; Sumner & Kinsella, 2021; Yıldırım & Arslan, 2020). Critically though, as these findings stem from an earlier phase in the COVID‐19 pandemic (Pappa et al., 2021) it is unclear whether resilience has retained its protective buffering effect throughout the pandemic.

Nevertheless, given COVID‐19 is a widely collective experience (Hirschberger, 2018) and HSCWs typically function within professional teams, the concept of team resilience bears relevance to adapting to COVID‐19 stressors. The importance of behavioural and psychological team cohesiveness in coping and maintaining well‐being, as well as collective efforts to learn, adapt to new ways of working and sharing ideas across the sectors (Barton et al., 2020) while dealing with work‐based stressors has been well documented (Meneghel et al., 2016; Stewart, 2010; Totterdell, 2000; West et al., 2009). Nevertheless, there has been a lack of empirical focus on the overall concept of team resilience within the context of COVID‐19 (Alliger et al., 2015; Greenberg et al., 2020). However, a small number of studies have reported on facets of team resilience, such as increased unity, higher morale and colleague social support as essential in dealing with the challenges faced by HSCWs during the pandemic (Khalili et al., 2021; Miotto et al., 2020; Vindrola‐Padros et al., 2020). Teamwork and solidarity across disciplines, as well as general social connectedness (Aughterson et al., 2021; Nitschke et al., 2021), has also been evidenced to reduce the effects of stress, burnout and improve well‐being during COVID‐19 (Aughterson et al., 2021; Norful et al., 2021). Thus, forming a rationale for further examination of the possible protective function of team resilience for HSCWs during COVID‐19. It is then also essential to understand mental health help‐seeking behaviour and work‐based supports (Shi et al., 2021; Tracy et al., 2020) used by HSCWs which may contribute to team resilience and help maintain or enhance their mental well‐being.

### 1.2 The current study

The ENACT study set out to explore the impact of COVID‐19 specific stressors (COVID‐19 perceived risks, worry, stress, burnout, PTSD) as well as protective factors (adaptive coping, team resilience, help‐seeking and work‐based supports) on HSCWs' mental well‐being during the third lockdown period. The study examined the work‐based supports that HSCWs most valued, and that were accessible to them, which may have helped them to maintain their mental well‐being and deal with COVID‐19 stressors. Guided by earlier work, we hypothesised that HSCWs with more COVID‐19 risk factors would have poorer mental well‐being and greater acute stress. We also hypothesised that HSCWs with more protective factors would have greater mental well‐being and lower acute stress. Given the unprecedented nature of the COVID‐19 pandemic and limited research that has specifically been conducted within the Scottish context, the study also included exploratory descriptive goals, using a multi‐method approach to analyses in order to support the development of hypotheses to be tested in future longitudinal studies in this area of investigation.

### 1.3 Scottish context

The current study was conducted during a period of significant political and social uncertainty. As a devolved nation the Scottish Government established five ‘protection’ levels of COVID‐19 restrictions setting out the rules that should be followed in each Local Authority area (Scottish Government, 2020). On the 26th of December 2020 mainland Scotland was moved to Level 4 restrictions to contain a new variant of the COVID‐19 virus. In Level 4, only essential shops and places of worship were allowed to open while up to 4 people from two households could meet outside and hospitality was closed. Extended households were permitted to support vulnerable people or those living alone. The first vaccines were offered from the 8th December 2020 while the first vaccines in care homes took place on the 14th December. Scotland went into full lockdown and a ‘stay at home’ order was re‐introduced on the 5th of January 2021. By the 10th of February one million people in Scotland had been vaccinated. Mid‐January brought suspension of all travel corridors and hotel quarantine was introduced. On the 22nd of February the country's youngest children returned to the classroom with all Primary school children returning on the 15th of March. The ‘stay at home’ order was lifted on the 5th of April 2021 with restrictions gradually being relaxed over the coming months.

It should be noted that HSCWs were operating under already difficult and stressful conditions even prior to the pandemic with a prolonged period of austerity measures since 2009 that resulted in cuts to public sector spending as well as changes in tax and welfare (Cavero & Poinasamy, 2013). Although the budget for the NHS was not reduced, a below average increase in funding alongside cuts in other areas of public spending, particularly in relation to social care had a significant impact (Kerasidou, 2019), including staff shortages and resource constraints across health and social care, all of which combined to create challenging working conditions for staff. These challenges were exacerbated by an increased demand for services as set out by Katikireddi et al., (2021) who argued that austerity measures disproportionately affected those already living in poverty, impacting both physical and mental health. During this time staff reported an increased focus on bureaucratic procedures, time‐keeping and gate‐keeping of services, within a context of diminishing services and resources impacting on the morale and motivations of HSCWs, resulting in moral distress and burnout (Grootegoed & Smith, 2018; Kerasidou et al., 2019). This was seen to conflict with the professional values of “caring” and relationship‐based practice, resulting in emotional dissonance. Further challenges to working conditions at this time were associated with the rise of new public management that led to the pay of pre‐dominantly female frontline staff failing to keep pace with counterparts in other sectors as well as cuts in pensions, sick pay and subsistence allowances (Baines & Cunningham, 2015). These challenges preceded the COVID‐19 pandemic, which have arguably further exacerbated the stressors facing HSCWs.

## 2 METHOD

The ENACT study adopted a mixed methods approach that involved an anonymous online survey using Qualtrics (2005) with a cross‐sectional design. Our study integrated a quantitative and qualitative analysis in the same study (Creswell & Plano Clark, 2011; Schoonenboom & Johnson, 2017). This approach is growing in popularity, due to its research advantages and opportunities: attaining a better understanding of the studied phenomenon, more robust empirical evidence and investigating novel and/or emerging phenomena (McCrudden et al., 2021; Taguchi, 2018). The survey captured quantitative data pertaining to HSCWs' responses to psychometrically valid measures of risks (e.g., on COVID‐19 stressors, burnout) and protective factors (e.g. adaptive coping, team resilience) to help illuminate understandings of the impact of COVID‐19 on their mental well‐being. Through including open ended free text questions relating to the impact of COVID‐19 on their mental well‐being and the supports they valued in helping them maintain their well‐being, the qualitative data helped capture HSCWs' own perspectives in terms of the challenges they faced and what helped them in adapting to these challenges. Adopting the use of mixed methods is a powerful tool for leveraging the strengths of one method to deal with the weakness of another (e.g., lack of participants' voices in quantitative methods) and to build a more comprehensive understanding of a study's conclusions in a specific context (DeCuir‐Gunby & Schutz, 2016).

### 2.1 Participants

The participants were recruited through convenience sampling. Inclusion criteria stated that participants had to be a HSCW working in Scotland during the COVID‐19 pandemic and aged 18 years old or over.

### 2.2 The survey

The survey included both closed and open‐ended questions exploring COVID‐19 risk and protective factors and their impact on mental well‐being. Questions relating to participants' demographical characteristics, working contexts and COVID‐19‐related worries were included.

The psychometric scales that were utilised in order to measure COVID‐19 risk factors were:

#### 2.2.1 COVID‐19 Perceived Risk Scale

Risk perception was measured using the COVID‐19 Perceived Risk Scale (Yıldırım & Güler, 2020), with questions relating to the emotional and cognitive dimensions of perceived risk. The 8‐item scale (e.g., ‘How worried are you about contracting COVID‐19?’) on a 5‐point Likert scale (1 = negligible, 5 = very large). A higher score reflects greater perceived risk of COVID‐19 when all of the items are scored to generate a total score. The Cronbach's alpha for this scale is 0.872.

#### 2.2.2 COVID‐19 Worry Scale

Respondents reported on their extent of worry about becoming infected, ill, losing financial stability or their job, inability to care for children or having children become infected, and inability to access necessary medications and food using a set of items adapted from the COVID‐19 Worry Scale used in a previous general population survey (Kolacz et al., 2020). Items were on a 4‐point ordinal scale (“Not worried”, “A little worried”, “Somewhat worried”, and “Very worried”). Though prior work has explored the factor structure of these items (Kolacz et al., 2020), individual items were used in order to compare amount of worry in individual domains.

#### 2.2.3 Coronavirus Stress Measure

COVID‐19‐related stress was measured using the Coronavirus Stress Measure (CSM; Arslan et al., 2020), which consists of five items (e.g., ‘In the last month, how often have you been upset because of the COVID‐19 pandemic?’) on a 5‐point Likert scale (1 = not at all, 5 = extremely). The items were summed to produce total scores, where a higher score was indicative of more COVID‐19‐related stress. The CSM has demonstrated to have strong internal consistency, with a Cronbach's alpha of 0.71 (Yildirim & Solmaz, 2022) to 0.83 (Arslan et al., 2020).

#### 2.2.4 COVID‐19 Burnout Scale

Burnout was measured using the COVID‐19 Burnout Scale (Yildirim & Solmaz, 2022). The 10‐item scale (e.g., ‘When you think about COVID‐19 overall, how often do you feel depressed?’) is on a 5‐point Likert scale (1 = never, 5 = always). A total score can be calculated by summing all items, with higher scores indicating higher levels of burnout. The Cronbach's alpha for this scale is 0.902.

#### 2.2.5 Post‐traumatic Stress Disorder Checklist

Acute stress was measured using a civilian version of an abbreviated form of the PTSD Checklist (PCL‐ 6; Lang & Stein, 2005). The PCL‐6 is a 6‐item scale (e.g., ‘*Difficulty concentrating’*) on a 5‐point Likert scale (1 = not at all, 5 = extremely). A score of 14 or over is indicative of clinically significant acute stress symptoms indicative of PTSD (Sudom, 2020). This scale was developed using two items from each of the three DSM‐IV symptom clusters. The Cronbach's alpha of internal consistency for this scale is α = 0.901.

The psychometric scales that were utilised in this survey in order to measure protective factors were:

#### 2.2.6 Brief Resilient Coping Scale

Resilient coping was measured (BRCS; Sinclair & Wallston, 2004) using a 4‐item scale that measures the tendency to cope with stress in a highly adaptive manner (e.g., ‘I look for creative ways to alter difficult situations) through a 5‐point Likert Scale (1 = does not describe me at all, 5 = describes me very well). A score of 4–13 is indicative of low resilient coping, 14–16 medium resilient coping and 17–20 high resilient coping. The BRCS has proven to be a valid and reliable measure of resilience (Mayordomo et al., 2020). The Cronbach's alpha of internal consistency for this scale is 0.815.

#### 2.2.7 Team Resilience Measure

Team resilience was measured using a 7‐item scale (Meneghel et al., 2016) based on Mallak's (1998) principles of organisational resilience. The items focus on perceptions of experiences, and the ability to perform adaptive behaviours (e.g., ‘In difficult situations, my team tries to look on the positive side’). The items are on a 7‐point Likert scale (1 = never, 7 = always), with higher scores representing greater team resilience. The Cronbach's alpha of internal consistency for this scale is 0.869.

#### 2.2.8 General Help‐Seeking Questionnaire

Help‐seeking was measured using 10 items (GHSQ; Wilson et al., 2005) on a 7‐ point Likert scale (1 = extremely unlikely, 7 = extremely likely). Items consist of possible people who could provide support, such as a mental health professional, partner or friend (e.g., *If you were having a personal or emotional problem how likely is it that you would seek help from the following people?*). The GHSQ evaluates the intent to seek help for general emotional problems. The measure has been found to be related to the search for current help during the last month and to predict future intention of seeking help (Olivari & Guzmán‐González, 2017).

The ENACT study sought to explore the impact of both COVID‐19 risk and protective factors on mental well‐being using the following measure:

#### 2.2.9 Short Warwick Edinburgh Mental Well‐being Scale

Mental Well‐being was measured using 7‐items (NHS Health Scotland, 2008; Tennant et al., 2007) is on a 5‐point Likert scale (1 = none of the time, 5 = all of the time). Statements relate to mental well‐being functioning (e.g., ‘I've been thinking clearly’). The item scores were combined before being transformed into metric scores (Stewart‐Brown et al., 2009), where higher scores denote better well‐being. This scale has demonstrated to be reliable with a Cronbach's alpha value of 0.86 (McFadden et al., 2021).

#### 2.2.10 Workplace supports

Participants were also asked whether they felt supported by their place of work, which work‐based supports they most valued, the availability of such supports and any other factors that they felt were important in terms of supports for their well‐being using open text responses. These responses were recorded through closed and open text questions.

### 2.3 Procedure

Following ethical approval from the University Ethics Committee, data collection commenced from the 23rd of December 2020 until the 31st of March 2021. An advertisement poster was circulated through social media (LinkedIn, Twitter and Facebook), NHS‐specific platforms and partner organisations to aid participant recruitment via an online link or advert QR code. All of the participants were presented with the inclusion/exclusion criteria, the objectives of the survey, the participant information sheet and a consent form that granted them the opportunity to decide whether to participate. They were informed that they were allowed to cease the survey at any point. Subsequent to completion, a debrief form was presented which included information on accessing support if needed. On average, the participants spent forty‐eight minutes (M = 48.61, SD = 39.08) completing the survey.

### 2.4 Analysis

The data was analysed using SPSS v26 software. The data was cleaned, missing data was inspected, and the standardised measures were scored. We first examined descriptive statistics to present relevant socio‐demographic and health information in relation to participant characteristics. Pearson's correlation was used to explore the association between COVID‐19 risk factors, protective factors and well‐being (see Table 1). Kurtosis and skewness scores and their cut‐off values were used to examine the assumption of normality (Blanca et al., 2013). Hierarchical regression analysis was used to explore the relationship among, and test hypotheses about, the impact on HSCWs' mental well‐being and levels of acute stress and COVID‐19 risk and protective factors. Significance level of *p* < 0.05 was used for all analyses.

**TABLE 1:** Correlation matrix of risk and protective factors for mental well‐being

Variable	*n*	M	SD	1	2	3	4	5	6	7
Well‐being	1341	19.69	3.53	1	−0.599**	−0.286**	−0.677**	−0.579**	0.427**	0.380**
PTSD	1361	14.54	5.97	−0.569**	1	0.425**	0.730**	0.630**	−0.286**	−0.244**
Risk perception	1337	25.13	6.26	−0.286**	0.425**	1	0.519**	0.494**	−0.160**	−0.137**
Burnout	1059	29.69	8.04	−0.677**	0.730**	0.519**	1	0.769**	−0.330**	−0.244**
Stress	1351	10.41	4.59	−0.579**	0.630**	0.494**	0.769**	1	−0.269**	−0.218**
Resilient coping	1340	11.48	3.71	0.427**	−0.286**	−0.160**	−0.330**	−0.269**	1	0.362**
Team resilience	1335	31.57	8.28	0.380**	−0.244*	−0.137**	−0.244**	−0.218**	0.362**	1

* p < 0.05

** p < 0.01

For the qualitative, open‐ended free‐text questions, content analysis of participants' comments was undertaken (see Krippendorff, 2018; Schreier, 2012). Initial descriptive codes were applied to participants' written responses. Subsequent text was compared to previously coded text and either allocated an existing code or provided a new one, thus grouping responses by similarity (Leech & Onwuegbuzie, 2007). Category development was guided by Vaismoradi et al. (2013). The first coder initially analysed the data, with the review being undertaken by the lead member of the research team, enabling both category refinement and research rigour. The researchers returned to the data several times during the analytical process to ensure that the results showed a strong connection to the analysed data. The categories of meaning (key codes) represented the highest level of abstraction for the reporting of the results. In the final phase, coded data were treated as variables for analysis conducted using descriptive statistics (frequency counts and percentages) in Microsoft Excel (Appendix S1).

Finally, the quantitative and qualitative data were integrated using a joint display of the quantitative and qualitative findings to emerge from the study as a framework for meaningfully making sense of the broader research findings (Plano Clark, 2019).

## 3 RESULTS

The participants were predominantly white, female, and degree‐level educated, aged between 18 to 69 (M = 27.2, SD = 11.6) and had worked a minimum of 6 months to 51 years (M = 16.5, SD = 11.2). Almost half of participants worked in the NHS (46.9%) followed by a local authority (25.8%). Participants were most likely to report that they worked with an adult population (36.1%). Table 2 outlines detailed demographic data.

**TABLE 2:** Participant demographics (*N* = 1364)

	*N*	%	M	SD
Age				
Under 30s	196	14.4	27.25	11.69
30s	264	19.4		
40s	374	27.4		
50s	435	31.9		
60s	94	6.9		
70+	1	0.1		
Gender				
Male	166	12.2		
Female	1178	86.4		
Other	20	1.5		
Sector				
NHS	634	46.9		
Local Authority	349	25.8		
Statutory	44	3.3		
Voluntary	83	6.1		
H&SC Partnership	44	3.3		
Other	124	9.1		
Multiple	75	5.5		
Education				
High School	137	10		
College	329	24.1		
University	844	61.9		
Other	54	4		
Years of work experience				
0–15	691	50.7	16.53	11.27
16–30	35.7	35.7		
31–45	181	13.3		
46–51	5	0.4		
Existing health problems				
Diagnosed mental health problem	246	18.1		
Long‐term health problem	152	11.2		
Other	65	4.8		
Multiple	145	10.7		
None	717	52.6		
Prefer not to say	36	2.6		
Had COVID 19‐related symptoms				
Previously had	395	29		
Had at time of survey completion	36	2.6		
No	930	68.3		
Been diagnosed with COVID 19				
Previously had	177	8.6		
Had at time of survey completion	15	1.1		
No	1228	90.3		
Able to work from home				
Yes	486	36.3		
No	634	46.5		
Sometimes	218	16		

*Note*: Percentages are valid percentages due to missing data.

### 3.1 Pre‐existing health conditions

43.8% of participants felt that previous episodes of physical and/or mental health difficulties had made the current COVID‐19 pandemic more difficult for them to deal with. Common health issues reported included anxiety, depression, asthma and acute stress. 5.3% of participants identified as being in the ‘shielding’ population.

### 3.2 COVID‐19 working context

Almost a third (31.4%) of participants felt that they had not been offered timely advice about how they should have responded to the COVID‐19 pandemic within their place of work. Over half (52.6%) of the participants had been able to work from home at least some of the time throughout the pandemic. Over 3/4 s (76.3%) of participants had experienced challenges with physical distancing at work.

### 3.3 Worries associated with COVID‐19

The most common worries reported by participants concerned fears about becoming infected with the COVID‐19 virus (88.4% at least a little worried, 20.2% very worried) and serious illness because of the COVID‐19 virus (84.7% at least a little worried, 22.9% very worried). Worries about reduced financial stability (56.4%), lack of access to necessary medication (45.9%), and lack of access to necessities were also common (43.8%).

### 3.4 Mental well‐being

The mean well‐being score for all participants was 19.6 (SD = 5.1), suggesting a low level of well‐being (score of 17 or less as indicative of depression). Analysis showed that lower well‐being scores were more common for participants with one or more of the following characteristics: younger and less experience in the sector; pre‐existing health condition(s); non‐binary or transgender; working in local authority sector; lower levels of education; unable to work from home and those that were unvaccinated. No statistically significant differences on mean well‐being scores were found between occupational groups (Table 3).

**TABLE 3:** Mean well‐being scores for occupational groups

Occupation	*N*	Mean	SD
Not given	10	18.81	2.64
Admin and office	72	19.15	4.14
Clinical role	76	19.20	4.08
Social and care workers	583	19.45	3.62
Nurse	304	19.67	3.23
Non‐clinical role	26	19.82	3.17
Management	35	20.34	3.33
Mental health role	37	20.52	3.50
Allied health professional	141	20.54	3.23
Doctor	57	20.58	3.29

### 3.5 Risk factors

The majority of participants were found to have high levels of COVID‐19 stress, burnout and risk perception scores and almost half of participants (49.3%) met the clinical cut‐off for acute stress (if symptoms persist risk of PTSD). Participants who reported having a diagnosed mental health problem and/or long‐term physical problem, were more likely to meet the clinical cut‐off for acute stress. Key signs of distress experienced by HSCWs were reduced energy levels, feeling that activities required greater effort, physical reactions (e.g., headaches), sadness, fear and anxiety. Only participants who reported no prior health problems in the last 5 years scored below the clinical cut‐off for acute stress (see Table 4).

**TABLE 4:** Risk and protective factors for mental well‐being

	Mean	SD
Risk factors		
COVID‐19 stress (high)	10.41	4.51
COVID‐19 burnout (high)	29.69	8.04
COVID‐19 risk perception (high)	25.13	6.26
PTSD (high acute stress)	14.54	5.97
Protective factors		
Resilient coping (low level)	11.48	3.71
Team resilience (low–moderate level)	31.57	8.28
Help‐seeking (moderate level)	34.39	8.76

Hierarchical regression analysis revealed that higher reported levels of COVID‐19 stress, burnout and risk perception predicted higher rates of acute stress (see Table 5); the results of the regression indicated that these three factors significantly accounted for 54.6% of the variance (*R* ^2^ = 0.546, *F* [3, 1031], 413.43, *p* < 0.001). That is, COVID‐19 stress (β = 0.157, *p* < 0.001), burnout (β = 0.584, *p* < 0.001) and risk perception (β = 0.157 *p* = 0.005) were all significant predictors of acute stress.

**TABLE 5:** Hierarchical regression analysis for COVID‐19 risk factors predicting acute stress

Variable	*B*	SE *B*	β	Sig.	Tolerance	VIF
COVID‐19 risk perception	0.048	0.024	0.049	*p* = 0.047	0.715	1.40
COVID‐19 burnout	0.435	0.025	0.584	*p* < 0.001	0.381	2.63
COVID‐19 stress	0.205	0.043	0.157	*p* < 0.001	0.400	2.50

### 3.6 Protective factors

The majority of HSCWs (68.9%) were found to score low on resilient coping (e.g., creative ways to alter situations, growing in positive ways). Almost a third (31.4%) felt that their colleagues were struggling to cope at work during the pandemic. HSCWs were found to have low‐moderate scores on team resilience. In terms of help‐seeking, HSCWs were significantly more likely to seek informal support (intimate partner, friend, parent, relative) as opposed to formal support (mental health professional, phone helpline, medical doctor) for dealing with personal or emotional problems (see Tables 4 and 6).

**TABLE 6:** HSCWs' mental health help‐seeking behaviour

Sources of help‐seeking	Mean score (SD)
Informal help‐seeking	Total mean score: 18.38
Intimate partner	5.38 (2.08)
Friend	5.28 (1.83)
Parent	3.72 (2.35)
Other relative/family member	4.0 (2.12)
Formal help‐seeking	Total Mean Score: 11.33
Mental health professional	3.42 (2.06)
Phone helpline	2.30 (1.71)
Medical doctor	3.98 (2.05)
Religious leader	1.63 (1.44)

*Note*: Each item on the scale was scored from 1–7, with 1 = Extremely Unlikely and 7 = Extremely Likely.

### 3.7 Most valued workplace supports

Given our interest in help‐seeking behaviour during the pandemic and the emerging findings that suggest that staff were more likely to seek informal rather than formal support, an in‐depth content analysis was carried out to better understand the reasons for this. Analysis was performed of participants' responses to the open‐ended question ‘What kind of workplace support, if any, has been most valuable during the COVID‐19 pandemic?’ Of the 1364 participants, 846 (62%) answered the question regarding what they considered to be the most valued workplace support that they had received. A total of 6 separate ‘categories of meaning’ (key codes) were developed (see Table 7).

**TABLE 7:** Descriptive summary of categories of meaning relating to most valued work‐based supports

Categories of meaning (key categories), number (%) of comments associated with category	Description of key category and examples of participant quotes
Peer support, *N* = 254 (30.3%)	Colleague/peer‐based support. Key aspects were colleague compassion and the idea of shared experiences and support (e.g. “Day to day conversations with colleagues. If's invaluable support to know that we are all experiencing this together—discussing our frustrations can be very therapeutic!!")
Workplace support, *N* = 189 (22.3%)	Support from workplace. Key aspects were facilities and services offered to support staff well‐being, and a culture of openness/communication around well‐being support for staff (e.g. “Good support available through weekly newsletters from HR on self‐help etc. Having access to the staff well‐being hub”)
Visible management and leadership, *N* = 120 (14.1%)	Sense of support from management and 'visible' leadership that communicates clearly and regularly with staff. Key aspects were accessibility of managerial support and recognition of individual staff needs (e.g. “Very regular meetings with colleagues and senior managers to have overview of service contingencies. Visible leadership that cares about staff”)
Team support, *N* = 116 (13.7%)	Being a part of a team and working together as a team. Moreover, team interaction was key, with regular interaction with team members valued. Sense of feeling supported within team (e.g. “Working closer together as a team to continue to provide a service for people”)
Safe working environment, *N* = 109 (12.8%)	Importance of PPE provision, prevention measures in the form of a COVID‐19 adapted workplace and practices. Additionally, the importance of COVID‐19 guidelines in place (e.g. “P.P.E and access to the vaccine with adjustments to how we work to keep safe during this challenging time”)
Communication, *N* = 58 (6.8%)	Importance of regular communication and being well informed. Communication using both face to face and online support resources and informed briefings (e.g. “Clear advice and regular communication about social distancing and control measures is essential”)

The most common category to emerge was that of (1) ‘Peer support’ (30% of all coded comments). This suggests a significant reliance on this informal source of support among HSCWs in dealing with COVID‐19 stressors. Further valued sources of support included (2) ‘Workplace well‐being supports’ including an open culture around well‐being and mental health help‐seeking, (3) ‘Visible management and leadership’ that were accessible and recognised individual staff and team based needs, (4) ‘Team support’ including regular interactions and joint working, (5) ‘Safe working environment’ in the form of COVID‐19 adapted workplaces, practices and guidelines and (6) ‘Communication’ that was clear, consistent and frequent between individual staff, teams and managers (with a clear leadership strategy). These findings highlight the importance of individual, teams, organisational and systems‐based supports for helping HSCWs deal with the challenges of COVID‐19 stressors and in maintaining their mental well‐being.

### 3.8 Perceived barriers to help‐seeking

It is important to recognise that as well as identifying valued sources of support, staff also pointed to challenges and barriers they experienced in accessing support. When asked about the availability of work‐based supports for maintaining their well‐being, 44.7% of participants reported that they had not received adequate support from their place of work (social, psychological, occupational or supervisory support). Inductive content analysis of the 180 participants (13.1%) who answered the open‐ended question ‘*is there anything else you consider important in terms of accessing support for your well‐being?*’ resulted in 4 categories of meaning (key codes). The key codes were: (1) ‘perceived stigma in mental health help‐seeking’ relating to concerns that seeking help for mental health issues will be negatively viewed by others and self, (2) ‘Fear of consequences of seeking help’ relating to fitness to practice and adversely impacting on job prospects, (3) ‘Insufficient time to care for self before others’ as caring professionals and (4) ‘Difficulty accessing supports needed’ due to excessive workloads and changes in working environment during the COVID‐19 pandemic (see Table 8). These findings highlight social, attitudinal and structural barriers to HSCWs seeking and accessing support for their mental well‐being during the third lockdown period.

**TABLE 8:** Descriptive summary of categories of meaning relating to accessing support in maintaining well‐being

Categories of meaning (key categories), number (%) of comments associated with category	Description of key category and examples of participant quotes
Perceived stigma in mental health help‐seeking, *N* = 49 (27.2%)	Concerns that seeking help for mental health issues will be negatively viewed by others and self (e.g. “I feel ashamed to ask for help”, “I put on a brave face for others or they'll think less of me”)
Fear of consequences of seeking help, *N* = 44 (24.4%)	Fear that to seek help for own mental health will lead to questions around fitness to practice. That seeking help might adversely impact on job prospects (e.g. “being expected to provide business as usual in a pandemic has had a major impact on my mental health. I'm scared to say how I really feel”)
Insufficient time to care for self before others, *N* = 44 (24.4%)	Being a care professional trained to provide care for others before self. Going beyond the line of duty in working role to help others during a personally challenging time (e.g. “it's been difficult looking after your own well‐being when you are concentrating on everyone else”)
Difficulty accessing supports needed, *N* = 43 (23.8%)	Accessing support for self as challenging due to excessive workload and changes in working environment during COVID‐19 pandemic (e.g. “I just do not have the time to get the help I need for my own mental health”)

## 4 INTEGRATIVE FINDINGS

**FIGURE 1:**
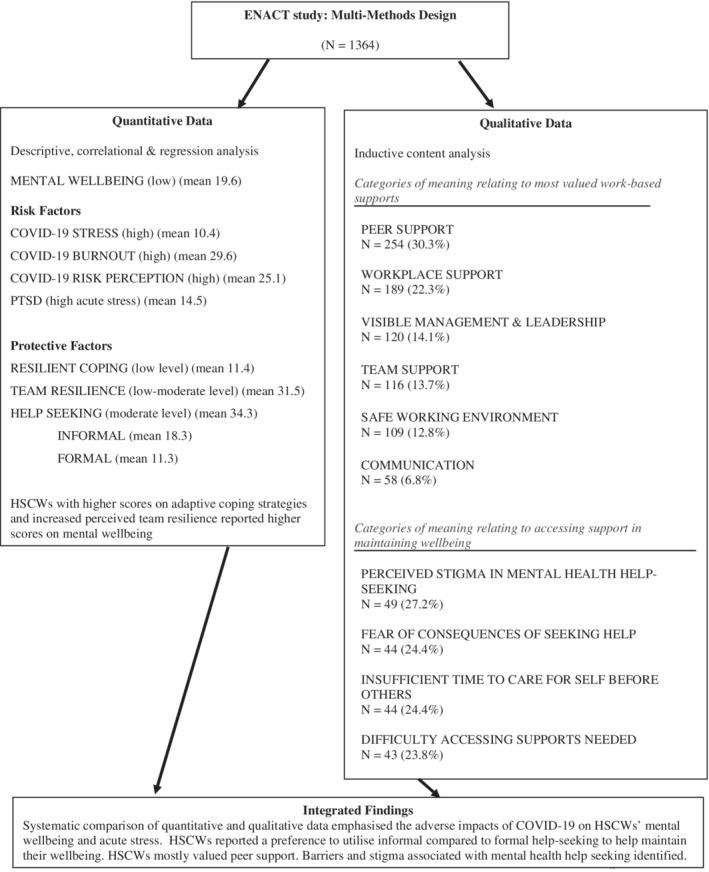
Integrative diagram of the quantitative and qualitative data.

The process of integrating the research findings involved systematically listing and comparing the qualitative and quantitative data in order to explicitly detail what each component added to the research area (Boeije et al., 2013; Bryman, 2006). Through the combined quantitative and qualitative results in accordance with APA reporting standards (Levitt et al., 2018), additional insights emerged (Creswell & Plano Clark, 2011). The quantitative data revealed that HSCWs were experiencing high levels of COVID‐19 stress, burnout, risk perception and acute stress (as indicative of PTSD if untreated). They were experiencing low levels of adaptive coping and low‐moderate levels of team resilience. They reportedly used moderate levels of help‐seeking and this was significantly more likely to be informal than formal. The qualitative data from the content analysis showed that HSCWs mostly valued peer support in terms of helping them maintain their mental well‐being. HSCWs also pointed to barriers to formal mental health help‐seeking including stigma and fear of the consequences of seeking help (see Figure 1). Together, these findings highlight the adverse impact of COVID‐19 stressors on the mental well‐being of HSCWs, the need for well‐being supports and to challenge some of the perceived barriers to help‐seeking.

## 5 DISCUSSION

The ENACT study explored the risk and protective factors impacting on the mental well‐being of HSCWs in Scotland during the third lockdown period of the COVID‐19 pandemic. The impact of COVID‐19 specific stressors (COVID‐19 perceived risks, stress, worry, burnout and PTSD) as well as protective factors (adaptive coping, team resilience, help‐seeking and work‐based supports) on HSCWs' mental well‐being were explored. The findings from our mixed methods approach to the analysis of data collected through our cross‐sectional online survey were comparable to recent studies conducted throughout the UK (BMA, 2020; Greenberg et al., 2021; Jordan et al., 2021; McFadden et al., 2021) and worldwide (Badahdah et al., 2020; Barzilay et al., 2020; Cag et al., 2021; De Kock et al., 2021; Fang et al., 2021; Feinstein et al., 2020; Inchausti et al., 2020; Mehta et al., 2021; Moitra et al., 2021; Rana et al., 2020; Vanhaecht et al., 2021; Young et al., 2021), reporting that HSCWs have experienced low levels of mental well‐being during the COVID‐19 pandemic. Lower mental well‐being scores were reported for HSCWs with one or more of the following characteristics: younger; less experience in the sector; pre‐existing health condition(s); non‐binary or transgender; working in a local authority sector; lower levels of education; unable to work from home and/or unvaccinated. No statistically significant differences in mean well‐being scores were found between occupational groups. Almost half of HSCWs (49.3%) met the clinical cut‐off for acute stress (if symptoms persist risk of PTSD). Higher reported levels of burnout and risk perception predicted higher rates of PTSD symptoms. These findings are comparable to recent work reporting low well‐being (McFadden et al., 2021), high levels of trauma symptoms (Greenberg et al., 2021; Greene et al., 2021) and high burnout rates (Pappa et al., 2021; Soares et al., 2022) among HSCWs.

Key signs of distress experienced by HSCWs associated with COVID‐19 were differences in energy levels, feeling that activities required greater effort, physical reactions (e.g., headaches), sadness, fear and anxiety. This aligns with previous research (Currie et al., 2020; Shanafelt et al., 2020), and suggests that key concerns contributing to high COVID‐stress include becoming seriously ill/infected, financial instability, difficulty accessing medicines and childcare issues (due to either home‐working or increased hours). In terms of concern over risk of infection, HSCWs with pre‐existing health conditions reported the pandemic being more difficult for them and they were more likely to meet cut‐offs for PTSD symptoms indicative of clinical significance. These findings are similar to those reported in general population studies (Alonzi et al., 2020; Vindegaard & Benros, 2020). An increasing body of research has demonstrated the susceptibility of HSCWs to developing mental health problems due to repeated exposure to work‐related traumatic events, along with the need to work under highly stressful circumstances (Canal‐Rivero et al., 2022; Carmassi et al., 2020; Mealer et al., 2009). The current pandemic has resulted in HSCWs being under both physical and psychological pressure increasing the risk of mental health sequelae (Krishnamoorthy et al., 2020). Given the high levels of acute stress found among HSCWs in the current study and the risks of trauma symptoms, the need for trauma informed practices in the workplace is essential in order to help prevent the likelihood of an exacerbation of such difficulties and worsening symptoms (Macedo et al., 2022; Morton et al., 2022). Indeed, this aligns with the National Trauma Training Programme (NTTP, 2021) for Scotland which has the ambition of a trauma informed and responsive workforce.

The fact that there were no statistically significant differences in terms of well‐being scores across occupational groups indicates that both health and social care workers, rather than specific organisations or working roles, have experienced the adverse impact of COVID‐19 stressors on their mental well‐being. Therefore, having accessible and robust access to psychological input and/or evidence‐based trauma interventions for those HSCWs that need it is essential (Carmassi et al., 2022). Peer support can be an important source of support and a useful means of sign‐posting to formal supports and interventions for those HSCWs that may benefit from such inputs. This is important given that almost a third of HSCWs reported that they had not been offered timely advice about how they should have responded to the COVID‐19 pandemic within their place of work. The findings suggest that HSCWs were significantly more likely to seek informal support as opposed to formal support for dealing with personal or emotional problems. Further research is needed to better understand these patterns of help‐seeking behaviour among HSCWs and to gain further insight into their preferences for drawing upon informal supports and peer networks and to better understand any perceived barriers in accessing formal support systems. Whether this preference is a product of perceived inadequacy of formal supports across sectors requires further investigation, as do other general help‐seeking patterns and formal support barriers (Richards et al., 2022).

While over four in ten HSCWs reported that they had not received adequate support from their place of work, those who did seek support most valued peer support, workplace supports, visible leadership and teamwork. The results point to the importance of peer, team, organisational and management support during stressful times and the need for frequent and transparent communication. This could include adaptive coping and communication skills training and team building events to equip teams to work and communicate effectively, particularly given new ways of working (Cogan et al., 2022; McFadden et al., 2021). HSCWs identified ‘barriers’ to accessing formal supports, including the perceived stigma in mental health help‐seeking, insufficient time to care for self before others and difficulty accessing supports needed. These findings are similar to previous work reporting that HSCWs fear that colleagues will judge them as professionally incompetent if they seek formal mental health support (Clement et al., 2015; Dearing et al., 2005; Galbraith et al., 2021; Walsh & Cormack, 1994). This finding is in line with existing research reporting that mental health‐related stigma, including that which exists within healthcare professions, creates significant barriers to accessing support (Ménard et al., 2022; Tay et al., 2018). It is paramount that approaches to combatting barriers related to mental health help‐seeking are prioritised by Health and Social Care Partnerships in order to prevent further deterioration of staff mental health and well‐being (Cogan et al., 2022; Knaak et al., 2017).

There were no statistically significant differences across occupational groups for protective factors (adaptive coping, team resilience or help‐seeking behaviours). The majority of HSCWs (68.9%) were found to score low on resilient coping (e.g. creative ways to alter situations, growing in positive ways). Comparable to earlier work (Deliktas Demirci et al., 2021), almost a third (31.48%) felt that their colleagues were struggling to cope at work during the pandemic. HSCWs were found to have low scores on adaptive coping, low‐moderate scores on team resilience and moderate scores on help‐seeking behaviours. Together, these findings suggest that demographic and situational/work‐based risk factors to mental health may be exacerbating one another and that protective factors for mental well‐being, in absence of organisation‐level support relative to developing these protective behaviours, were low to moderate for HSCWs during the third lockdown period.

Whether individual resilience fosters team resilience or vice versa remains an empirical debate worthy of further research (see Alliger et al., 2015; Southwick et al., 2016). However, these findings create a baseline upon which to develop hypotheses for longitudinal research which seeks to ascertain whether interventions that seek to increase protective factors to help buffer the adverse impact of COVID‐19 stressors are effective. They also provide a basis on which to recommend interventions based on both individual and team‐based factors in attempts to assist HSCWs in coping with stressors, both personal and systemic, rather than focusing on either factor alone. Growing evidence shows that reinforcing social bonds among colleagues, collaborative working and building effective and cohesive teams are highly protective factors in maintaining and improving staff well‐being (Aughterson et al., 2021; Cogan et al., 2022; Greenberg & Tracy, 2020; Khalili et al., 2021; Pink et al., 2021). Management and leadership initiatives in mental health services should be targeted at creating this combination within the working environment and prioritising staff well‐being. While our findings support the employment of efforts to instil a trauma informed approach to improve both individual and team resilience, it is important to recognise that any benefits will be limited in a wider context of understaffing and under resourcing (Hiam et al., 2020; Lasater et al., 2021).

### 5.1 Strengths and limitations

One of the strengths of the current research is that it utilised psychometrically valid COVID‐19 specific measures of stress, worry, burnout and risk perception, therefore, provides more specific outcomes indicative of risk factors for mental well‐being associated with this pandemic. To our knowledge, this was the first study to specifically explore the impact of both risks and protective factors of COVID‐19 on the mental well‐being of HSCWs in Scotland and to explore which workplace supports were valued and accessible to them. Secondly, the survey was inclusive of those working in both health and social care sectors and, therefore, provides a broader perspective than research which has largely focused on healthcare professionals only. Thirdly, the inclusion of open‐ended free‐text questions provided rich and in‐depth responses relating to workplace supports valued by HSCWs. A free form response gave participants the opportunity to respond openly without pre‐determined responses proposed by researchers. This provided richer detail around what work based supports had been helpful or valuable to HSCWs. Finally, to understand the best way to support HSCWs in Scotland, we have gained these findings from the perspectives of those working at a particularly challenging time; the third lockdown period. However, this study is cross‐sectional, therefore, present a snapshot into understanding the impact on HSCWs. There is a need for longitudinal research to better understand the long‐term impacts of COVID‐19 on the mental well‐being of HSCWs during the recovery phase of this pandemic (Cunningham & Pfeiffer, 2022). There is also a need for future work to capture the experiences of diverse HSCWs (e.g. ethnic minorities, LGBT+, economically disadvantaged and protected characteristics) and across geographical locations (e.g. urban versus rural) given the emerging research evidence this health and social inequalities experienced by specific groups of HSCWs (Hussein, 2022).

## 6 CONCLUSION

The ENACT study provides important transferable insights into the impact of COVID‐19 on the mental well‐being of HSCWs in Scotland during the third lockdown period; our findings will be of relevance to HSCWs working across diverse socio‐cultural contexts. As Scotland has moved from the response to the recovery phase of the COVID‐19 pandemic, continued support for those working in health and social care settings is paramount. Understanding the impact of the COVID‐19 pandemic should not be limited to exploring risk factors but should extend to the influence of protective factors which have the potential to buffer the negative impact on HSCWs' mental well‐being. Further longitudinal work is needed in order to understand the long‐term risks and protective factors for HSCWs' mental well‐being moving forward. Such work will help inform our understandings of how best to support HSCWs through developing bespoke psychosocial interventions that aim to help reduce stress, burnout and trauma. Gaining a better understanding of barriers to mental health help‐seeking, seeking to increase adaptive coping skills and finding ways to build team resilience is essential. While the benefits associated with interventions aiming to improve HSCWs' well‐being are likely to have a positive impact on patient outcomes, this will be limited in a wider context of understaffing and under resourcing within the health and social care sector. Maintaining and improving staff well‐being requires a multidimensional approach involving individuals, teams and the wider organisation/working environment.

## AUTHOR CONTRIBUTIONS

All authors contributed to the study conception and design. Material preparation and data collection were performed by all authors. Data analysis write up was performed by Chloe Kennedy, Zoe Beck, Lisa McInnes, Jacek Kolacz and Nicola Cogan. All authors contributed to the final manuscript, which all authors read and approved.

## FUNDING INFORMATION

This work was supported with funding from the Scottish Government.

## CONFLICTS OF INTEREST

The authors have no relevant financial or non‐financial interests to disclose.

## ETHICS STATEMENT

Ethical approval was granted by the University Ethics Committee, University of Strathclyde. This study was performed in line with the Declaration of Helsinki.

## CONSENT

All participants provided informed consent to participate in the study.

## Supporting information


**Appendix S1** Supporting Information

## Data Availability

The data can be made available to reviewers upon request.
