# Supplementary material for: ENACT study: What has helped health and social care workers maintain their mental well‐being during the COVID‐19 pandemic?
Source: Health Soc Care Community. 2022 Sep 6:10.1111/hsc.13992. Online ahead of print. doi: 10.1111/hsc.13992 (PMC9539329; doi:10.1111/hsc.13992)
Supplement: Supplementary file 1 — Appendix S1 Supporting Information [file HSC-9999-0-s001.docx]

Supplementary file

ENACT Study: TH**E** IMPACT OF COVID-19 O**N** THE MENT**A**L WELLBEING OF HEALTH AND SO**C**IAL CARE WORKERS in Scotland: A MIXED-ME**T**HOD STUDY

**Missing data**: Data was initially obtained from 2041 participants overall. However, 106 did not provide informed consent, 284 did not continue after providing informed consent and 287 did not complete all the measures included in the survey. After removing data from these participants, 1364 participants remained and their responses were used for analysis.

Table not included within the manuscript but as supplement to reported findings:

**COVID worry scale: frequency count of responses to each item**

|  | **Number and frequency count of responses for each scale point** | | | | |
| --- | --- | --- | --- | --- | --- |
| **How worried are you that you will…** | **Not worried (1)** | **A little (2)** | **Somewhat (3)** | **Very (4)** | **N/A** |
| **Be infected with the COVID-19 virus** | 0.12  (n = 158) | 0.37  (n = 511) | 0.31  (n = 418) | 0.2  (n = 276) |  |
| **Be less financially stable?** | 0.44  (n = 593) | 0.27  (n = 368) | 0.15  (n = 204) | 0.14  (n = 197) | 0.001  (n = 1) |
| **Be unable to care for your child/children?** | 0.62  (n = 849) | 0.13  (n = 173) | 0.11  (n = 147) | 0.11  (n = 152) | 0.03  (n = 42) |
| **Be unable to get necessary medications?** | 0.54  (n = 737) | 0.23  (n = 309) | 0.16  (n = 219) | 0.01  (n = 98) |  |
| **Become infected by your child/children if they attend school?** | 0.67  (n = 911) | 0.13  (n = 174) | 0.01  (n = 134) | 0.01  (n = 93) | 0.01  (n = 51) |
| **Become seriously ill because of the COVID-19 virus?** | 0.15  (n = 205) | 0.35  (N = 471) | 0.27  (371) | 0.23  (n = 312) | 0.01  (n = 4) |
| **Lose your job?** | 0.67  (n = 917) | 0.16  (n = 224) | 0.2  (n = 134) | 0.06  (n = 86) | 0.001  (n = 2) |
| **Be unable to get important necessities (e.g., food)?** | 0.56  (n = 763) | 0.25  (n = 346) | 0.13  (n = 172) | 0.06  (n = 79) | 0.002  (n = 3) |
